# Tribological Behavior of a Rubber-Toughened Wood Polymer Composite

**DOI:** 10.3390/polym13132055

**Published:** 2021-06-23

**Authors:** Valentina Mazzanti, Annalisa Fortini, Lorenzo Malagutti, Giulia Ronconi, Francesco Mollica

**Affiliations:** Department of Engineering, University of Ferrara, 44122 Ferrara, Italy; annalisa.fortini@unife.it (A.F.); lorenzo.malagutti@unife.it (L.M.); giulia.ronconi@edu.unife.it (G.R.); francesco.mollica@unife.it (F.M.)

**Keywords:** wood polymer composites, tribology, wear, toughening agent, pre-treatments, water absorption

## Abstract

Wood polymer composites or WPCs are increasingly used as substitutes for natural wood in outdoor applications due to their better environmental sustainability and the consequent reduction in carbon footprint. In this paper, the presence of an elastomer used as a toughening agent (Santoprene by Exxon Mobil) in a polypropylene-based WPC containing 50 wt % wood flour was investigated in terms of its tribological behavior by dry sliding wear tests. These were performed after two environmental pre-conditioning treatments, i.e., drying and water soaking. The ball-on-disk configuration under a constant load was chosen along two sliding distances. Dynamic mechanical thermal analyses were used to reveal the effect of the toughening agent on the storage modulus and damping factor of the composites. Results in terms of weight loss measurement and coefficient of friction were obtained, together with surface morphology analysis of the worn surfaces at the scanning electron microscope and 3D profilometer. An abrasive wear mechanism was identified, and it was shown that the toughening agent improved wear resistance after both pre-treatments. This beneficial effect can be explained by the increase in strain at break of the WPC containing the elastomer. On the other hand, the water soaking pre-treatment produced severe damage, and the loss of material cannot be completely compensated by the presence of the toughening agent.

## 1. Introduction

In the last decade, the interest in wood polymer composites (WPC) as cheap and environmentally friendly materials has increased more and more, both in the academic community and industry. These bio-composites are constituted by a thermoplastic matrix, such as polyethylene (PE) or polypropylene (PP), and wood particles or fibers coming from wastes of industrial or agricultural origin [1,2,3]. The main advantages of these materials have reduced the use of petroleum-derived resources, are low density, and have reasonable mechanical properties. In fact, the presence of the natural filler improves the strength and stiffness of the polymeric matrix, thus making WPCs suitable for mildly structural applications, such as in the automotive industry, in civil engineering, and also as acoustic absorbing materials [4,5]. Furthermore, the hydrophobic matrix can partially protect the hydrophilic natural filler from humidity, making usage in marine and outdoor environments also possible [6,7].

On the other hand, recent literature studies highlight that wet environments can become aggressive on WPCs, and this is particularly significant when the percentage of wood is equal to or exceeds 50 wt % [8,9,10,11]. In fact, the hygroscopic wood particles have a strong tendency to absorb water and swell, despite the hydrophobic nature of the polymer. Since the wood filler is anisotropic, the polymer–wood interface can be subject to significant stresses that can lead to its failure, thus causing the composite to lose its mechanical integrity [8]. Another critical disadvantage of WPCs is brittleness: also in this case, the chemical incompatibility and the poor interfacial adhesion between wood and polymer can explain low toughness and limited strain at break [12].

Both hygroscopicity and brittleness can be reduced by improving the fiber–matrix interface. Usually, this can be achieved through an adequate coupling agent [13,14] or an appropriate chemical treatment performed on the natural fibers [15]. Both strategies create a chemical bond between matrix and filler; improve the reinforcement dispersion; and, as a result, lead to a better interface. Interestingly, some extra benefits can also be obtained by the addition of a suitable toughening agent [16]. This additive, usually an elastomer, acts by encapsulating the wood particles and creating a tougher interface with the polymer matrix. When this action mechanism becomes effective, brittleness is decreased at the price of a certain stiffness reduction [17]. In the literature, many toughening agents have been studied with WPC systems, such as ethylene–propylene rubber (EPR) [14,18,19,20,21], ethylene–propylene–diene rubber (EPDM) [12,22,23], styrene–butadiene rubber (SBR) [24], ethylene–octene copolymer (EOC) [25,26], and styrene–ethylene–butylene–styrene (SEBS) [12,27]. In general, the results have shown that despite a large decrease in strength and stiffness, the presence of a toughening agent is effective in reducing brittleness and improving strain at break and impact properties. Moreover, the presence of a toughening agent can also limit the negative effect of degradation induced by water absorption [16].

Common applications of WPC boards as decking and flooring materials have prompted the recent research attention towards the tribological properties of these materials [28,29,30,31,32,33]. Generally speaking, the wear behavior of fiber reinforced composites is a complex phenomenon, not fully studied and understood. However, all authors agree that the presence of the wood fibers is beneficial in terms of wear properties [34,35,36] and that wear resistance increases with the natural filler content [29,37]. In fact, the coefficient of friction (COF) and the wear rate (WR) of WPCs are lower if compared with the neat polymer matrix or with a synthetic fiber reinforced composite [38]. In addition, COF decreases further as the wood fiber content increases [36,37]. Interestingly, the presence of an appropriate coupling agent can reduce the material volume loss [39], and the same result can be obtained by using an appropriate natural fiber treatment [40], because in both cases, the fiber–matrix adhesion is improved.

In terms of the authors’ knowledge, there are no papers dealing with the influence of toughening agents on the tribological properties of WPC. The present paper aims at evaluating the effects of the presence of a 20 wt % commercial thermoplastic elastomer (Santoprene, Exxon Mobil) on the tribological properties of a PP-based WPC containing 50 wt % wood flour in terms of dry sliding behavior. The influence of two different environmental pre-conditioning treatments has also been evaluated, mainly because of WPC common applications in wet environments that cause a decrease in their mechanical properties. The tribological tests have been conducted by a standard ball on disk configuration, using a steel ball as a counterpart, and considering two different sliding distances. The surface morphology of the wear tracks has been observed through scanning electron microscopy (SEM) and 3D non-contact profilometric analysis to identify the wear mechanisms.

## 2. Experimental Procedures

### 2.1. Materials and Samples Preparation

The materials considered in this study were two blends of a 70 wt % PP-based WPC (PP CO 68/BZ) and a 30 wt % PP-based WPC (PP 30 S) purchased from Plasticwood s.r.l. Mazzantica di Oppeano, VR, Italy, and a PP-based thermoplastic vulcanizate (Santoprene 201-55 from ExxonMobil, Irving, TX, USA), i.e., a TPV. All constituents were blended in such a way that a fixed wood content equal to 50 wt % was obtained for both composites. In particular, one material contains no toughening agent and is indicated simply with “WPC”, while the other one contains 20 wt % of TPV and is identified as “t-WPC”. The two compositions and their theoretical densities are listed in Table 1. Slabs of 2 mm thickness and 50 mm width of the composites were obtained with a single screw extruder (P.R.T. Service and Innovation, S. Agostino (FE), Italy) possessing a screw diameter of 50 mm and a length over diameter ratio of 40. The extruder was equipped with a venting zone to remove moisture and volatiles and with a breaker to improve mixing and compaction. Before extrusion, all natural filler containing pellets were dried at 80 °C for 15 h. A flat temperature of 190 °C was set along the extruder barrel, while the die temperature was maintained at 180 °C. The complete mechanical characterization of WPC and t-WPC can be found in [41,42], respectively.

### 2.2. Dynamic Mechanical Thermal Analyses

Dynamic mechanical thermal analysis (DMTA) was conducted in double cantilever mode using Tritec 2000 DMA (MP Strumenti, Bussiero, Italy). Specimen dimensions were 30 mm × 5 mm × 2 mm and were obtained from extruded profiles. Dynamic temperature scans were made from room temperature up to 170 °C, with a heating rate of 2 °C/min and 1 Hz frequency. The storage modulus E’ and the damping factor tan *δ* were measured as a function of temperature. 

### 2.3. Hardness and Roughness

Hardness measurements were carried out by a DGTS (Milano, Italy) hardness tester, in agreement with the ASTM D2240 standard. The mean Shore D hardness values were calculated from five indentations. The roughness of the samples was evaluated before wear testing through a Talysurf CCI-Lite non-contact 3D profilometer (Taylor-Hobson, Leicester, UK).

### 2.4. Environmental Pre-Treatments

A set of 24 slabs were cut to an 80 mm length to obtain the specimens, which were dried for 16 h at 80 °C in an air-circulation oven, reaching about 0.3% moisture content, and then weighed with an analytical digital balance possessing 0.1 mg resolution (Mettler Toledo AE240, by Mettler Toledo, Columbus, OH, USA). Half of these samples were also environmentally conditioned in distilled water at room temperature for 24 h and then weighed again. All of them absorbed a quantity of water equal to about 3.7%. These two pre-treatments were labeled as dry and wet, respectively.

### 2.5. Tribological Tests

Wear tests were performed in dry condition at room temperature using the Ducom TR-20LE tribometer (Ducom INSTRUMENTS PVT LTD, Bangalore, India) in a ball-on-disk configuration. A 100Cr6 steel ball (EN-ISO 683-17, AISI 52100), 10 mm in diameter, was used as a counterpart. Tests were carried out in reciprocating mode with a frequency of 2 Hz and an applied load of 10 N. The tests were run at a constant linear sliding speed of 0.13 m/s and total sliding lengths of 300 and 600 m for both WPC and t-WPC. Three replicas for each condition were performed. The evolution of COF was acquired and registered during the tests. The wear rate (WR) was evaluated by gravimetric analyses; hence, before and after each tribological test, the samples were weighed with an analytical balance (Mettler Toledo AE240, by Mettler Toledo, Columbus, OH, USA).

### 2.6. Analyses of the Worn Surfaces

The appearance of the worn surfaces at the end of the wear tests was first investigated by camera detections through a mirrorless digital camera (Canon EOS M6, Canon, Tokyo, Japan) with Tokina 100 mm lens (Tokina, Machida, Japan). To further investigate the topography of the samples after tribological tests, we analyzed the worn surfaces by a non-contact 3D profilometer (Talysurf CCI-Lite, Taylor Hobson Precision, Leicester UK). Five cross-sections of the wear tracks were used to estimate the dimensions of the wear profile for all materials and pre-treatment conditions. The wear tracks were also analyzed with a Zeiss EVO MA15 (Zeiss, Oberkochen, Germany) scanning electron microscope. SEM analyses were conducted on gold-sputtered coated samples at an accelerating voltage of 18 kV. SEM images were recorded in secondary electron imaging (SEI-SEM) mode.

## 3. Results and Discussion

### 3.1. Hardness and Roughness

Table 2 summarizes the hardness and roughness values of the WPC and t-WPC samples in their initial conditions. Although the average value of the t-WPC roughness was greater than the WPC roughness, the difference was not statistically significant, and their values were comparable. On the other hand, the presence of the toughening agent reduced the hardness of the t-WPC composite, as expected.

### 3.2. Dynamic Mechanical Thermal Analyses (DMTA)

**Figure 1:**
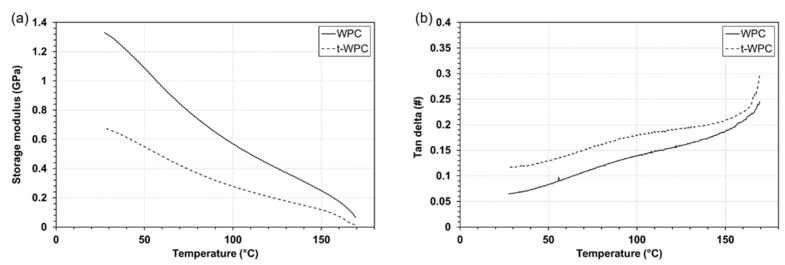
(**a**) Storage modulus and (**b**) damping factor vs. temperature for WPC and t-WPC.

The results of DMTA are pictured in Figure 1. The storage modulus of both materials decreased with temperature with the same qualitative behavior. From the quantitative point of view, WPC was about twice as much as t-WPC at all temperatures (Figure 1a). This was clearly due to the presence of the toughening agent, which seemed to be well dispersed within the material. On the other hand, the damping factor increased with temperature within the whole range that was observed. The peak at 165 °C was related to the melting temperature, while the smaller peak at around 100 °C, which was visible for both composites, was also observed in [41] and was related in that paper to the melting of some PP crystallites. These results confirm that t-WPC is a more dissipative material than WPC.

### 3.3. Analysis of the Worn Surfaces

**Figure 2:**
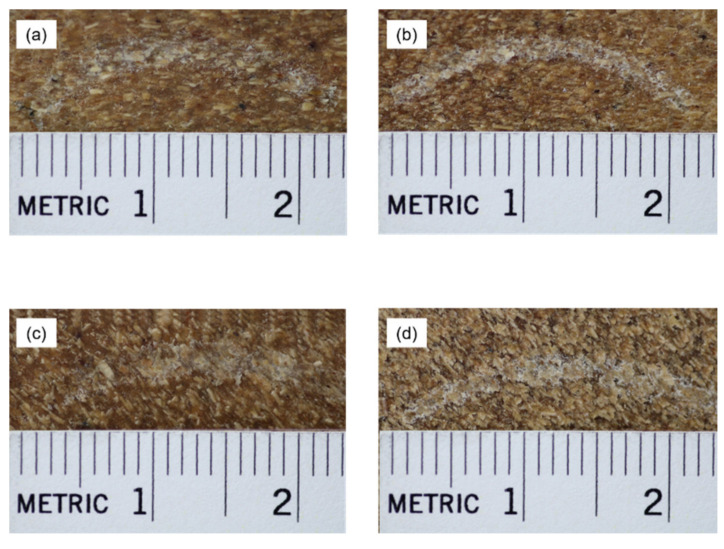
Representative images of the worn surfaces 600 m sliding distance for (**a**) dry condition t-WPC, (**b**) dry condition WPC, (**c**) water-soaked preconditioned t-WPC, and (**d**) water-soaked preconditioned WPC.

The representative images of the worn surfaces after 600 m of sliding distance for t-WPC and WPC in both pre-treatment conditions are presented in Figure 2. The wear traces are well evident, and a few differences can be appreciated. The water-soaked samples (Figure 2c,d) showed a wider and more clearly visible trace compared with the dried samples (Figure 2a,b), while the WPC traces (Figure 2b,d) appeared thinner than those of the t-WPC samples (Figure 2a,c). Material whitening was quite evident in the wider trace of the toughened samples, and this can be justified by the plastic deformation caused by the steel ball, while the water-soaked samples seemed to have undergone higher wear.

### 3.4. Profilometric Analysis

**Figure 3:**
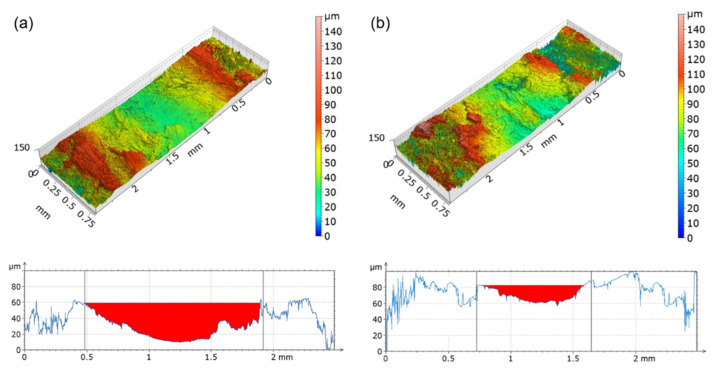
Representative 3D view and 2D cross-sectional profiles of the 600 m sliding distance worn surfaces after drying pre-treatment for (**a**) t-WPC and (**b**) WPC.

**Figure 4:**
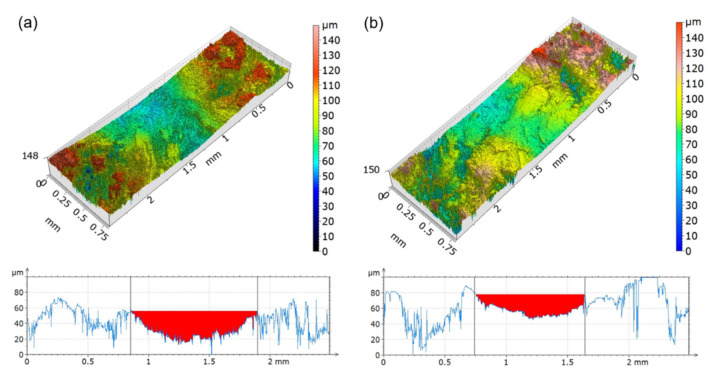
Representative 3D view and 2D cross-sectional profiles of the 600 m sliding distance worn surfaces after water soaking pre-treatment for (**a**) t-WPC and (**b**) WPC.

In order to obtain more information about the wear behavior of the composites and a precise quantification of the trace dimensions, we analyzed a surface portion of 0.8 mm × 2.5 mm enclosing the wear trace with an imaging profilometer. This analysis was significant because the roughness of all materials was similar (Table 2). The results for all materials and preconditioning treatments are shown in Figure 3 and Figure 4, while in Table 3, the average measurements of the width and the depth of the wear traces are listed.

Dried t-WPC exhibited deeper and larger wear traces compared with those of WPC (Figure 3 and Table 3). Moreover, the WPC samples showed a higher dispersion, which could be only partly explained by a more difficult measurement of the wear trace. Indeed, the wear surface of this material appeared rougher, and the wear traces were less pronounced, despite the starting roughness values of the WPC and t-WPC samples being comparable (Table 2). Even after the water soaking pre-treatment (Figure 4 and Table 3), t-WPC showed deeper traces, but the difference between the two composites seemed to be less significant, and this could have been due to the presence of water absorbed on the surface during the test. Comparing the two different pre-treatments (Figure 3 and Figure 4 and Table 3), we found that the values that were obtained seemed to indicate that for t-WPC, the effect of water soaking in terms of the depth of the trace was very limited and not effective on the shape of the wear traces. On the other hand, the soaking pretreatment performed on the WPC composites seemed to increase damage, although not many additional considerations can be made because the standard deviation was very high.

### 3.5. Weight Loss and Wear Rate 

**Figure 5:**
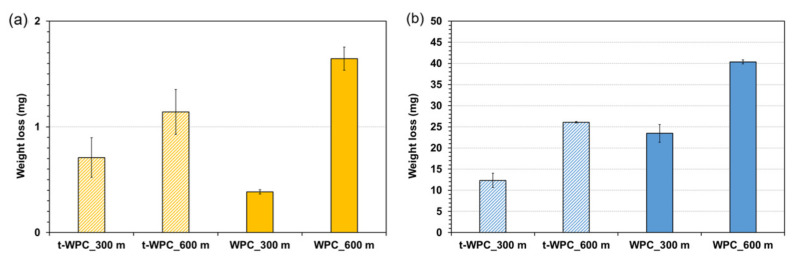
Weight loss for all material and sliding distances tested after (**a**) dry pre-conditioning and (**b**) water soaking preconditioning.

Weight loss measurement is another useful way to evaluate the wear behavior, and the results of this measurement are pictured for all materials and testing conditions in Figure 5 and are listed in Table 4. As expected, the weight loss increased with increasing sliding distance, and this was obviously due to the progressive removal of material with distance. In particular, the dried t-WPC weight loss increased by about 60% as the sliding distance doubled, while the increase relative to the dried WPC was about 330%. This can be an indication that the wear mechanism of the two materials was different. The situation changed when the composites were subjected to the water soaking pretreatment. Here, the damage mechanism was probably more similar because the weight loss increment at a double distance was about 110% for t-WPC and about 75% for WPC. 

**Figure 6:**
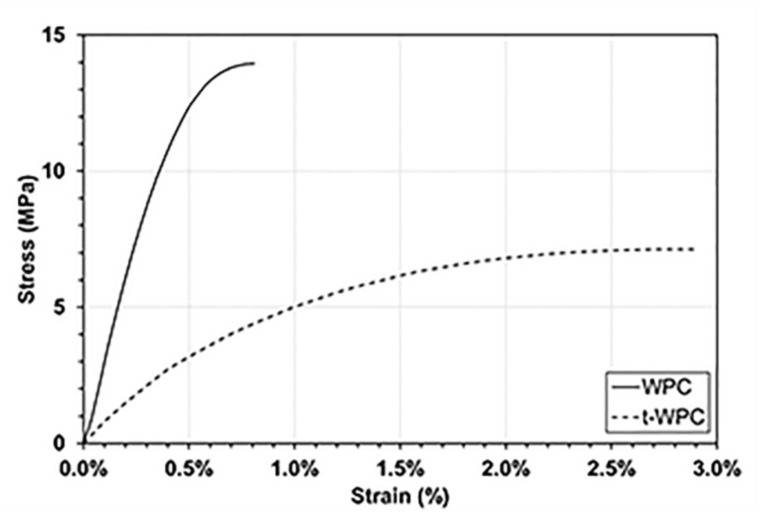
Stress vs. strain in tension for WPC and t-WPC composites, as taken from [42].

Concerning the weight loss values, all materials subjected to the water soaking pre-treatment showed higher weight loss values compared to the dry samples, and t-WPC showed a reduced amount of lost material compared with WPC, especially at 600 m sliding distance. These results were statistically significant, both after 300 m and 600 m sliding distance (ANOVA, *p* < 0.001). This last result was quite surprising. In fact, a limited loss of material measured after the tribological test would correspond to better wear resistance, but these results did not seem to be consistent with those obtained with the profilometer (Figure 3 and Figure 4). This was particularly evident when comparing Figure 3a,b with the second and fourth columns of Figure 5a. This discrepancy can be justified by the type of measurement that is being carried out, as profilometry readings can be affected by the mechanical properties of the material that is being analyzed as explained next. In Mazzanti et al. [42], both composites were characterized in tension following the ISO 37-2011 standard, and the results in terms of stress vs. strain plot are pictured in Figure 6. The t-WPC showed a more ductile behavior with a greater strain at break and lower strength and stiffness than WPC. This was in agreement with the hardness measurements reported in Table 2, as well as the DMTA measurements displayed in Figure 1a. As a consequence, the steel ball had a penetration that was higher on t-WPC than WPC during the tribological test, and this produced a larger plastic deformation. For this reason, a wider and deeper wear trace measured with the profilometer may not correspond to a greater quantity of removed material. On the other hand, a more brittle material, such as WPC, can more easily undergo superficial brittle fractures and material loss.

The same trend can be observed in Figure 5b, which shows the water-soaked samples. In this case, the t-WPC seemed to resist wear better than WPC. On the other hand, the presence of the toughening agent was not sufficient to compensate for the damage due to water soaking, as severe damage to the material caused by this pre-treatment can be appreciated. The presence of water absorbed within the material surface during the tribological tests led to an increase in the weight loss by an order of magnitude compared with the dry pretreatment samples. In fact, the effects of water damage on the mechanical properties of the material are well known [8], and this phenomenon also seems to extend to the wear properties. This can be explained by the swelling of the wood particles that decreases the strength of the wood–polymer interface, favoring material detachment and fiber pull out. 

The wear rate values for both materials and pre-treatments are listed in Table 4. The sliding distance seemed to have had no effect on the wear rate of both materials after dry pre-conditioning. This means that the material was mainly removed in the first part of the test, i.e., within the first 300 m, while doubling the distance, the wear rate values decreased when both bio-composites were tested after the water soaking preconditioning.

### 3.6. Friction Coefficient

**Figure 7:**
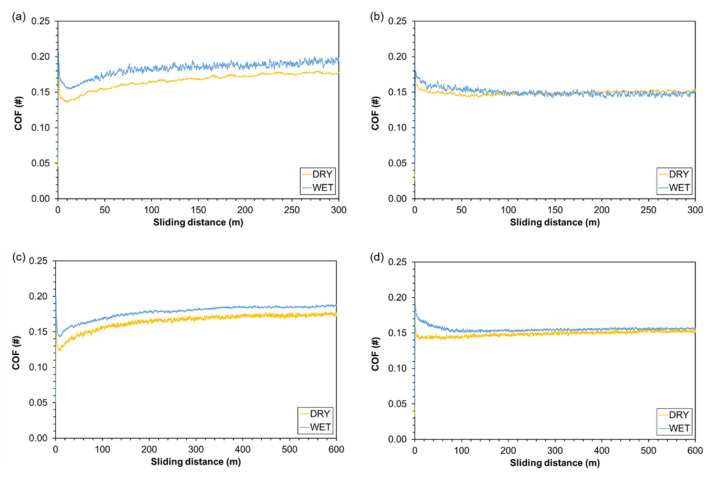
Coefficient of friction versus sliding distance in dry and wet preconditioning: (**a**) t-WPC, 300 m; (**b**) WPC, 300 m; (**c**) t-WPC, 600 m; (**d**) WPC, 600 m.

The curves of COF vs. sliding distance for both bio-composites and pre-treatments are displayed in Figure 7. The pre-treatments had a limited effect on COF, in particular, the dry t-WPC had slightly lower values compared with the samples that were soaked in water (Figure 7a,c), while for WPC, the difference between the two curves was negligible (Figure 7b,d). Moreover, the t-WPC curve displayed COF values that were slightly higher than those of WPC. This difference can be explained because the presence of the toughening agent makes t-WPC more compliant: the steel ball penetrates to a greater depth, and since the friction force between two bodies is proportional to the effective contact area, the tangential force developed during the test increases. Another important difference can be found in the curve at the beginning of the test. In the case of WPC, the COF had a monotonous decreasing behavior generated by the transition from static friction to kinematic friction, reaching a stable plateau after about 150 m (Figure 7b,d), which amounted to 0.150 (±0.003) for dry conditions and 0.157 (±0.002) after soaking pretreatment. The situation was different for t-WPC, in that after the decreasing trend related to the change from static to kinematic friction, the t-WPC curve showed a minimum before increasing and reaching a plateau after about 300 m (Figure 7a,c). In this case, the plateau values were 0.165 (±0.002) for dry preconditioning and 0.178 (±0.001) after soaking pretreatment. All COF differences are statistically significant, as confirmed by ANOVA (*p* < 0.001).

A justification of this behavior can be found in the composition of the toughening agent: Leblanc [43] analyzed this commercial grade of Santoprene, performing a xylene extraction to quantify the percentages in weight of the various components. In detail, Santoprene contains EPDM (48.51 wt %), PP (5.64 wt %), and 45.85 wt % of lubricant oil. This significant quantity of oil is meant to act as a processing aid, i.e., an additive that migrates towards the surface of the material during extrusion, thus creating a lubricant layer. This presence of oil favors a COF decrease in the first part of the test, then when the surface, and thus also the lubricant, are abraded, the COF begins to increase again. Lubricating agents are common additives in WPC formulations to improve wall slip and processability, but the quantity of this processing aid is never more than 3–5 wt % [44].

### 3.7. Wear Mechanisms

**Figure 8:**
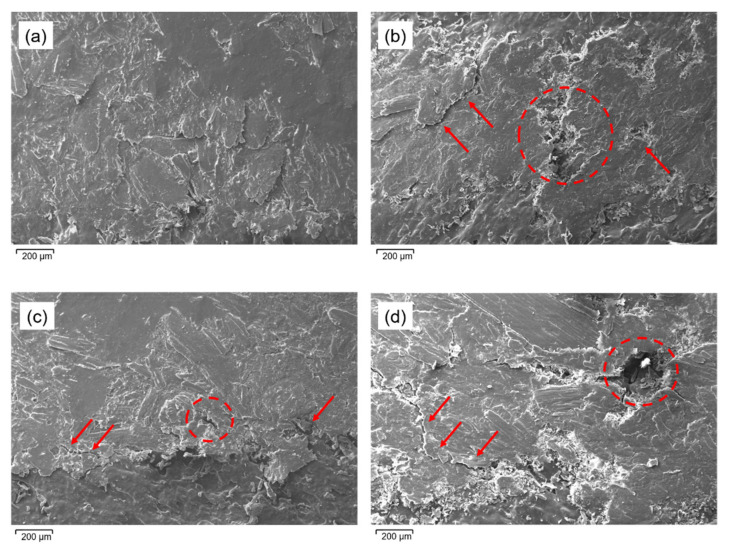
SEM images of the wear traces of (**a**) t-WPC dry condition; (**b**) WPC dry condition; (**c**) t-WPC wet condition; (**d**) WPC wet condition.

In Figure 8, the SEM scans of all composites after 600 m of sliding distance are shown. Comparing the two materials, a difference in terms of the morphology of the wear trace can be appreciated. The t-WPC (Figure 8a,c) after both pre-treatments showed a generalized plastic deformation, in agreement with the previous results of the profilometric analyses. The material was more ductile, and during the tribological test, the polymer matrix can deform plastically without fracturing. The situation was different in the case of WPC since the absence of the toughening agent made the material stiffer but also more brittle, and during the wear test, the polymeric matrix broke at the surface. This is evident in Figure 8b,d, as shown by the indications in red.

Abrasive wear is typical of bio-composites, as reported in [32,34,37,39]. This is caused by four effects that occur sequentially: polymer matrix deformation, fibers wear, breakage and pull-out of fibers, and fiber–matrix interfacial debonding under the form of micro-cutting. In detail, abrasion is due to fragmented fibers, formation of debris, that are removed from the bio-composite by the steel ball, and the mechanism is more severe when the difference between the tested material and the counterpart is larger. This wear mechanism is made worse in polymers because the heat generated in the contact region cannot be easily dissipated, leading to temperature increase and thereby softening the material even further [38,40].

## 4. Conclusions

In this work, the wear resistance of a 50 wt % PP-based WPC with and without the addition of a toughening agent was investigated. Wear was studied after two environmental pre-treatments, i.e., after drying and water soaking, and an abrasive mechanism was identified. In general, the presence of the toughening agent increased wear resistance compared with a non-toughened WPC in terms of mass loss. This benefit was probably due to an increase in the strain at break, i.e., a reduction in brittleness. On the other hand, the presence of water absorbed by the wood particles on the material surface made both composites very sensitive to wear, and the presence of the toughening agent, although beneficial, did not completely counteract this damage. The coefficient of friction did not depend too much on the environmental pre-treatments, while in the first part of the test, the character of the COF curve was modified by the presence of the lubricant contained in the toughening agent.

## Figures and Tables

**Table 1:** Bio-composite compositions.

Material	Wood (wt %)	PP (wt %)	TPV (wt %)	Theoretical Density (g/cm^3^)
WPC	50	50	0	1.07
t-WPC	50	30	20	1.09

**Table 2:** Hardness and roughness for WPC and t-WPC.

Material	Ra (µm)	Hardness (Shore D)
WPC	13.9 ± 2.2	28.9 ± 1.0
t-WPC	17.6 ± 3.0	24.7 ± 0.9

**Table 3:** Worn traces dimensions obtained with profilometer analysis.

Material	Pre-Treatment	Width (µm)	Depth (µm)
WPC	DRY	0.9 ± 0.2	26.6 ± 9.5
	WET	1.3 ± 0.3	37.8 ± 18.9
t-WPC	DRY	1.8 ± 0.5	49.9 ± 5.5
	WET	1.7 ± 0.6	48.6 ± 6.5

**Table 4:** Weight loss and wear rate for all materials and conditions.

Material	Pre-Treatment	Sliding Distances (m)	Weight Loss (mg)	WR (mm^3^/Nm)
WPC	DRY	300	0.39 ± 0.03	0.004
	WET	300	23.5 ± 3.6	0.094
	DRY	600	1.65 ± 0.1	0.002
	WET	600	40.4 ± 0.8	0.043
t-WPC	DRY	300	0.71 ± 0.2	0.003
	WET	300	12.3 ± 3.0	0.084
	DRY	600	1.1 ± 0.2	0.002
	WET	600	26.1 ± 0.3	0.038

## Data Availability

Publicly available datasets were analyzed in this study. These data can be found here: https://www.researchgate.net/publication/352645869_Tribological_Behavior_of_a_Rubber-Toughened_Wood_Poly-mer_Composite_data, accessed on 10 May 2021.
